# Civil Service Examination for Physicians

**Published:** 1891-05

**Authors:** 


Civil Service Examination for Physicians.—An open competitive examination of candidates for Junior Assistants and

Female Physicians in the State Hospitals for the Insane, will be held by the State Civil Service Commission at the Capitol, in Albany, June 11th. Candidates must be residents of the State, and must have had one year's hospital experience or three years' experience in the general practice of medicine.

